# From planning to execution: Interactive virtual-reality assisted craniotomy planning in meningioma surgery

**DOI:** 10.1016/j.bas.2026.106102

**Published:** 2026-05-22

**Authors:** Sebastian Lehmann, Martin Vychopen, Alexandru Guranda, Florian Wilhelmy, Ferdinand Weber, Johannes Wach, Erdem Güresir

**Affiliations:** Department of Neurosurgery, University Hospital Leipzig, Leipzig, 04103, Germany

**Keywords:** Mixed reality, Virtual reality, Augmented reality, Meningioma, Approach planning, Craniotomy

## Abstract

**Introduction:**

In meningioma surgery, extent and accuracy of the craniotomy are vital to achieve the predefined extent of resection. We routinely integrate interactive virtual-reality (iVR) assistance into craniotomy planning.

**Research question:**

Are preoperatively iVR-planned approaches transferable to craniotomies subsequently performed in the operating room?

**Material and methods:**

We examined 83 patients surgically treated with meningiomas, where the approach strategy was preoperatively planned and demonstrated using the MagicLeap 2 or HoloLens 2. The iVR-planned approach (iVR-Group) was compared to the real craniotomy (Real-Group) by location and relative size of craniotomy, as well as position and number of burr holes.

**Results:**

When comparing the craniotomy size, the mean anterior-posterior (AP)-ratio was 0.40 in iVR-group vs 0.41 in Real-group with 17.5% mean deviation, while the mean lateral ratio was 0.45 vs 0.49 with 15.9% mean deviation.

In 9.6% of procedures, a diverging number of burr holes were used, in 3.6% the position was altered from iVR-group to Real-group. In testing for equality (TOST-test) both AP and lateral-ratio deviation showed significant results: AP-ratio-deviation: p = 0.015, CI: −0.047-0.013, equivalence margin ±0.059; lateral ratio-deviation p = 0.041, CI: −0.068-0.004, equivalence margin ±0.071. The geometrical index was 0.423 for iVR group and 0.448 for Real-group. The percentage deviation between both groups was 5.9%, indicating high transferability of preoperative planning.

**Discussion and conclusion:**

With state-of-the-art iVR-planning of meningioma surgery approaches, craniotomies respecting pathological and anatomical relations and conflicts can be accurately simulated. IVR planning may mark a new level of interactive learning and strategic planning in neurosurgery.

## Highlights

• Interactive Virtual Reality enables patient specific 3D craniotomy approach planning.
• 83-case study shows stong match of iVR-planned vs real craniotomy for craniotomy size + burrholes
• Geometric deviaion between iVR plan and surgical approach was below 6%.

## Abbreviations

AR: Augmented Reality
VR: Virtual Reality
MxR: Mixed Reality
AP: Anterior-posterior
SD: Standard deviation
SSS: Superior Sagittal Sinus
GM: Geometrical mean

## 1 Introduction

Meningiomas are the most common intracranial tumors and surgical resection represents the therapy of choice (Ogasawara et al., 2021). Even more so than in the case of deep-seated lesions, the precise and meticulous planning of the craniotomy is essential given their primarily superficial origin, in order to achieve the intended surgical objective.

In order to reach the predefined extent of surgical resection, the extent of craniotomy must expose the tumor itself, as well as dural tail, while allowing the surgeon to choose an optimal trajectory for manipulation and resection, taking surrounding eloquent neuronal or vascular structures into account. When a carefully planned craniotomy is effectively executed, the surgeon can achieve the intended extent of resection according to the Simpson grading system, thereby reducing the risk of recurrence.

Until recently, preoperative approach planning primarily relied on two-dimensional depiction of CT or MRI-scans, supported by anatomical textbooks, craniometric points and simple measurement tools such as rulers (Graziano et al., 2025). Although the introduction neuronavigation has significantly facilitated the planning of surgical approaches, the improvement in visualization - and the associated learning curve – have remained largely confined to the surgeon and their assistant during each individual procedure.

The emergence of mixed-reality (MxR) technology has introduced a novel planning modality that enables neurosurgeons to incorporate patient-specific anatomy and pathological features into their preoperative strategy (Mahvash et al., 2017). This technology enables stepwise, three-dimensional planning of the procedure prior to any intervention taking place in the operating room (Bova et al., 2013; Colombo et al., 2023).

According to current literature, the use of MxR shows an increasing role with applications ranging from training, planning of surgical procedures to the implementation into the operating room and rehabilitation (Mishra et al., 2022; Durrani et al., 2022). In practical application, MxR can be subdivided into purely virtual environments (VR) and augmented reality (AR). Both environments enable interaction to a predefined degree.

In other high-responsibility work fields like aviation, extensive simulator training is a mandatory element of training before entering the cockpit (Human Factors in Aviation, 2010; RWL German Flight Academy, 2025; Simulation in Aviation Training, 2016). In Neurosurgery, however, experience is mainly gathered during practical application in the operating room. To reduce procedural risks while simultaneously enhancing the learning curve of residents in a safe and controlled environment, we propose the integration of interactive VR-based (iVR) simulator training, complemented by plenary discussion, into routine neurosurgical practice.

Currently, we practice iVR-supported surgical approach planning on a daily basis, before applying AR-neuronavigation using the identical volumes intraoperatively.

Neurosurgical approaches are prepared by surgeons or residents and presented to the entire team through interactive mixed-reality visualization. The ensuing discussion of feasibility or alternatives enables experienced team members to contribute their expertise, while fostering collaborative learning among junior colleagues.

In this study we aim to assess the accuracy and transferability of these mixed reality-based surgical planning by comparing the preoperative planned approaches with those executed intraoperatively in meningioma surgery.

## 2 Methods

In this study we investigated the preoperative iVR-presentation and surgical approach planning for 83 cases of meningioma surgery. Using data from preoperative CT and MR-Imaging, 3D-models of the skin, skull, brain, tumor and other relevant structures were 3D-moleded using the Brainlab Smart Brush volumetric function (Brainlab, Feldkirchen, Germany). The craniotomies in meningioma surgery encompass both standardized approaches for skull base tumors as well as tailored, freehand approaches for convexity meningiomas.

Several augmented reality headsets were developed for commercial use. At our institution, two devices were eligible for daily use: Microsoft's HoloLens 2 (Guo and Prabhakaran, 2022) and the Magic Leap 2 (Curtis). Both headsets work slightly different, when rendering alterable three-dimensional models from the medical image files. While Microsoft's HoloLens 2 utilizes externally pre-rendered 3D models, the Magic Leap 2 can directly interact with the Brainlab-software and 3D-planning, providing an elegant and time-saving alternative (Moon and Barua, 2024).

The iVR-presentation was performed either via the HoloLens 2 or via MagicLeap 2. When using the HoloLens 2, rendering was performed using the publicly available FreeCAD software (*Fr*eeCAD Project, *Version 0.21.2*. https://www.freecad.org/). The presenter could freely choose between both headsets.

**Fig. 1:**
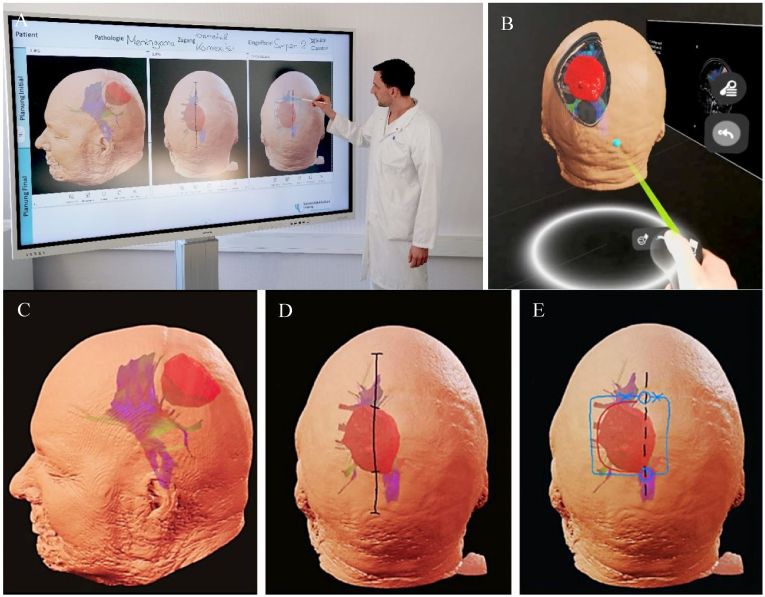
Depiction of MxR-presentation for approach planning in case of a parasaggital meningioma in plenary setting. A: Presentation of the approach and verbalization of the strategy; B/C: Positioning and depiction of relevant structures in iVR-setup. D: Marking of planned skin incision; E: Marking of planned craniotomy and dural opening.

The planning was interactively presented by the surgeon or assistant who later performed the surgery via on of the iVR-platforms. Projection onto a shared screen allowed all team members to follow the planning process (see Fig. 1). The presentation followed structured protocol.

1. Positioning of the 3D-head model, which simulates the positioning of the head in Mayfield Skull-Clamp.
2. Virtual demonstration of the skin incision, burr-hole(s), craniotomy and durotomy and marking those on previously obtained screenshot.
3. Stating the exact steps of the tumor-resection (e.g. devascularization if feasible, debulking and capsule resection with respecting of critical anatomical structures).
4. Stating of planned Simson grade.
5. Team discussion of the proposed plan, refinement as needed, and final determination of the surgical procedure

**Fig. 2:**
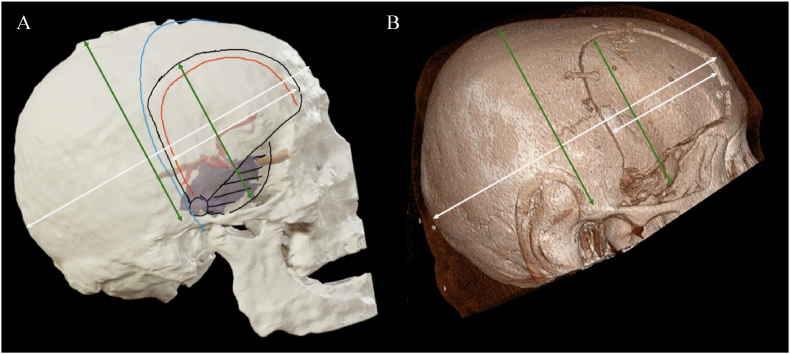
Depiction of measurements creating AP-ratio (white) and lateral ratio (green) in planned (A) vs. real craniotomy (B).

For our analysis, the preoperatively planned craniotomy was compared to the craniotomy performed on the respective patient. For comparison, either 3D skull depictions from postoperative CT or MR Images were used to investigate the type and position of the craniotomy. Because the rendered models were not consistently depicted in scale, the craniotomy size was evaluated by the lateral and anterior-posterior (AP)-ratio of skull to craniotomy in axial or sagittal projection (see Fig. 2). In cases of postoperative MRI depictions, craniotomy dimensions were evaluated based on T2-wheighted signal enhancement of the bony defect. AP-ratio was determined by measuring the maximum AP diameter of both the skull and the corresponding craniotomy. For the lateral ratio, anatomical landmarks such as the zygomatic root or the mastoid tip were used as a reproducible reference point that define the inferior boundary. Measurement conditions were kept identical for each pair of approaches (iVR and real) to ensure comparability. Additionally, the number and position of burr holes were compared.

**Fig. 3:**
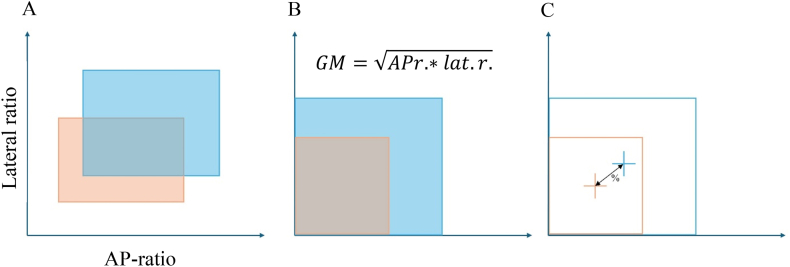
Depiction of lateral and AP-ratio (A), the geometrical mean (GM) = $\sqrt{AP-ratio*lateral\;ratio}$ (B), as well as the percentage deviation of central tendency (C).

For descriptive analyses, SPSS (IBM Corp. Released, 2023. IBM SPSS Statistics for Windows, Version 29.0.2.0 Armonk, NY: IBM Corp) was used for statistical analysis. A normal distribution of the data was confirmed by the Shapiro-Wilk test. Paired t-tests and “two one-sided t-tests” (TOST) equivalence test for AP-ratio and lateral ratio were performed in Excel (Microsoft Corporation, Redmond, WA, USA). A deviation of approximately 1 cm between craniotomy planning and real procedure was defined to be an acceptable accuracy. Our group showed an average head AP-diameter of 154 mm. Calculating with a mean AP-ratio of 0.41 and lateral ratio of 0.49, the equivalence margin was set to 0.059 for AP-ratio TOST-test and to 0.071 for lateral ratio. To address the area based on ratios of the craniotomy, the geometric mean was calculated by taking the square root of AP and lateral ratio. This reflects the central tendency of a craniotomy in varying shapes in order to adequately reach the underlying pathology using the ideal approach pathway. Subsequently, percentage deviation of central tendency was calculated to quantify the planning precision (see Fig. 3).

## 3 Results

**Fig. 4:**
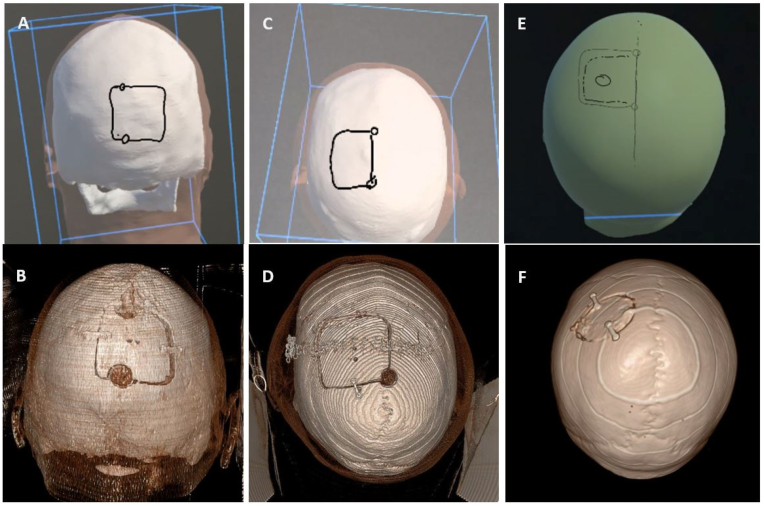
Depiction of planned vs. real craniotomy, A + B: correctly planned craniotomy, C + D: discordant number of burr holes, E + F: discordant craniotomy.

In this study, 83 patients who underwent surgical treated for meningiomas from 2024 to 2025 were evaluated for position and size of craniotomy, as well as placement and number of burr holes (see Fig. 2). The meningiomas were predominantly located supratentorially at the convexity (37%), as well as falcine (18%) and at the sphenoid wing (15%). Frontobasal meningiomas at the olfactory groove (6%) and at the sphenoid plane or tubercle (6%), as well as posterior fossa meningiomas at the petrous bone (3.6%), petroclival (3.6%) were included. 2.4% of meningiomas showed predominantly intraosseous growth, while another 2.4% arose from intraventricular (see Table 1). Four procedures addressed recurrent tumors. The approach and location used intraoperatively corresponded with the meningioma position and iVR-planning in all cases. However, involvement of the superior sagittal sinus (SSS) differed between iVR-gounp and Real-group in two cases (see Table 2). One relevant alteration in craniotomy shape was observed (1.2%), in which the initial planning intended a squared craniotomy involving the (SSS), while the respective real craniotomy was performed in a parasagittal round fashion (see Fig. 4, E/F).

**Table 1:** Localizations of treated meningioma.

Localisation	
Convexity	31 (37.3%)
Falcine	15 (18.1%)
Sphenoid Wing	14 (16.9%)
Frontobasal	3 (3.6%)
Sph. Plane/Tub. Sph.	5 (6.0%)
Olfacotry Groove	5 (6.0%)
Petrous bone	3 (3.6%)
Petroclival	3 (3.6%)
Intraventricular	2 (2.4%)
intraossary	2 (2.4%)

**Table 2:** Approach chosen.

Approach	
Convexity parietal	30 (36.1%)
Convexity involvin SSS	17 (20.5%)
Pterional	29 (34,9%)
Retrosigmoid	5 (6.0%)
Combine infra/supratent.	2 (2.4%)

Deviation of approach	2 (2.4%)

Comparing the setting of burr holes, no alteration was observed for standard craniotomies like pterional, retrosigmoid and suboccipital. In both groups, one burr hole was consistently used for lesions reached via retrosigmoid, pterional approaches or being located at the convexity without requiring sinus-involving craniotomy (See Table 3). For falcine or parasagittal meningiomas, the set of one vs. two burr holes was divergent in 7 cases (8.4%), one combined infra/supratentorial approach involved the transverse sinus diverging from the iVR-planning (1.2%).

**Table 3:** Comparison of burr hole setting in planned presentation vs. real surgical treatment.

Burr hole(s)	Plan	Reality
SSS	27 (32.5%)	27 (32.5%)
Zygoma	31 (37.3%)	31 (37.3%)
Convexity	16 (19.3%)	17 (20.5%)
Trans. Sinus	2 (2.4%)	3 (3.6%)
Asterion	7 (8.4%)	7 (8.4%)

1 Burr hole	62	61
2 Burr holes	21	22

Diverging number		8 (9.6%)
Diverging Position		3 (3.6%)

**Fig. 5:**
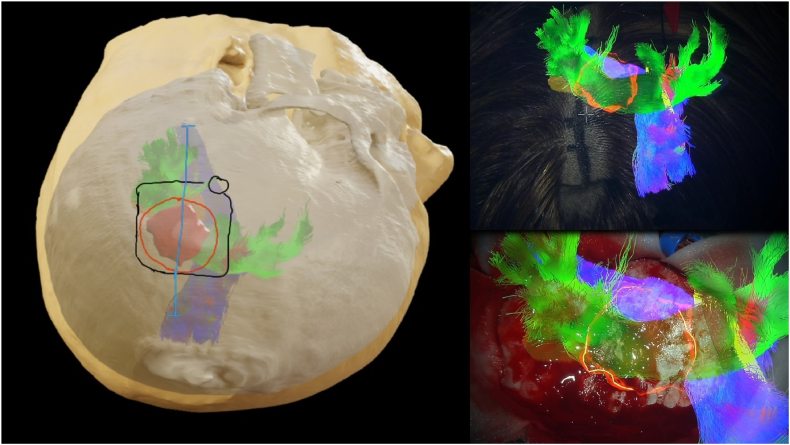
Deviation of the AP-ratio (A) and lateral ratio (B) in MxR-group to Real-group in percentage.

The mean AP- ratio for the iVR-group was 0.39 with SD of 0.11, compared to a mean of 0.41 with a SD of 0.12 in the Real-group. The mean deviation between iVR-group and Real-group was 17.5% with a SD of 19.8% (see Fig. 5A).

The mean lateral ratio for the iVR-group was 0.46 with a standard deviation (SD) of 0.14, compared to a mean of 0.49 with a SD of 0.14 in the Real-group. The mean deviation between iVR-group and Real-group was 15.9% with a SD of 20.1% (see Fig. 5B).

To evaluate equivalence of the iVR-group and Real-group in TOST-testing, the equivalence margin was set to 15% of mean AP- and lateral ratio. Both ratios showed to be significantly equivalent; AP-ratio-deviation: p = 0.015, CI: −0.047-0.013, lateral ratio-deviation p = 0.041, CI: −0.069-0.0043 (see Table 4).

**Table 4:** TOST (*two one sided tests*) equivalence test for lateral ratio, equivalence margin: 15% (≈1 cm) of AP-ratio deviation = ±0.059; Lateral ratio deviation = ± 0.071).

TOST: AP-ratio deviation		
	t	p-values
Upper	2,29548	0,01149
Lower	−4,15187	0,00003
	Max	0,01149
**Equivalence shown.**		**p = 0,0115**

TOST: Lateral ratio deviation		
	t	p-values
Upper	1,74872	0,04111
Lower	−4,66597	0,00000
	Max	0,04111
**Equivalence not shown.**		**p = 0,0411**

The geometrical index was 0.423 for iVR group and 0.448 for Real-group. The percentage deviation between both groups was 5.9%.

## 4 Discussion

Our study shows that craniotomies to treat meningioma preoperatively planned in an iVR setting are suitable to accurately simulate the operative reality allowing the surgeon to perform a reliable stepwise planning. We demonstrate a valid transferability between model and reality by position and number of burr holes, as well as position and size of craniotomy.

In current state, the application of MxR in neurosurgery can be divided in three main applications: Teaching (Paro et al., 2022), surgical planning and navigation (Winkler et al., 2024; Grunert et al., 2024; Olexa et al., 2024).

1. Teaching

A survey conducted at our institute revealed the great interest for AR to be used for teaching medical students, as it helps understanding complex anatomical relations and surgical approaches (Wach et al., 2024). Enhancement of hands-on courses and simulation training using MxR was favorably endorsed and showed to increase the trainees' satisfaction (Silvero et al., 2023; Malhotra et al., 2025). First attempts to integrate VR-simulation training into a curriculum were perceived as highly realistic and increased skill and confidence (Neyazi et al., 2025). However, we still lack a curriculum-based routine application of VR into training of students and residents. Our approach demonstrates how iVR-planning of operative approaches to meningiomas may be used for training of students and residents. When performed in a plenary setting, a wide range of pathologies and surgical approaches can be demonstrated. The subsequent discussion provides an opportunity for experienced team members to share their expertise in approach strategy, while less experienced team members can also actively and passively contribute, creating a learning effect for all participating.

2. Planning

For the planning of neurosurgical procedures, first studies using 3D Holograms described an increased anatomical understanding without impact on the preparation time (Colombo et al., 2023). Zanuttini et al. reported sufficient use of MxR-planning of thalamic tumors to increase understanding in complex anatomical situations (Zanuttini et al., 2025). First transfer of cranial VR planning to AR-use in the operating room was sufficiently performed in 2023 (Jean et al., 2024). Practical use was shown by Najera et al. (2024), who used MxR in a series of complex AVMs to visualize deep arterial feeders. Tonchev et al. (2025), reported experience integrating AR-planning into the microscope's head-up display while Kos et al. (2023) highlight an increase of efficiency and teamwork between surgeons and nonsurgical staff in presence of MxR. With rapidly evolving technology, MxR in different variations can be expected to be an integral part of the neurosurgical armamentarium in near future.

In our routine, planning and teaching is closely related, as on a daily basis, an iVR-presentation is followed by a plenary discussion to create deep understanding and creating transparency regarding surgical approach strategy and decision-making. In planning of meningioma surgery, an approach is tailored to the respective pathology and its surrounding eloquent vascular and neurological structures. This optimal approach may follow a standard pathway like for most skull-base meningioma (Moscovici et al., 2016; Elhammady et al., 2012), as well as being freely positioned, shaped and adapted to the tumor's location in case of convexity, falcine or parasagital meningioma. The iVR approach-simulation is suitable for evaluation of both, as the benefits and limitations of standard approaches, as well as free approaches can be visualized to preoperatively decide if they are feasible for the pathology.

In planning a free approach, key aspects like different positioning, altering the point of access, as well as the trajectory to pathology are evaluated, visualized and dynamically adapted to create the optimal approach. In our cohort, no discrepancies in craniotomy position, as well as number and position of burr holes were observed in standard approaches. For free craniotomies, the main discrepancy observed is the usage of dual vs. a single burr hole in craniotomies involving the SSS in preoperative simulation. There are predictable factors that influence the decision to place a single or multiple burr holes, such as performing a craniotomy directly over or crossing a sinus. However, factors like adherent dura mater or sinus injury may lead to unplanned placement of additional burr holes. This divergence may also be attributed to a different evaluation of the possible complication of a SSS injury (Samadian et al., 2011; Benjamin et al., 2019) Still, we observed high accuracy of the simulated craniotomy extent regardless of its position with both, AP and lateral ratio reaching significant equivalence. Regarding the geometrical mean analysis, the observed 5.9% percentage deviation indicates robust alignment between the virtually planned approach and the real craniotomy. Within the context of neurosurgical precision, such a marginal discrepancy supports the clinical reliability of advanced preoperative planning. We believe a carefully considered and accurately performed approach is a key to achieve the best possible degree of resection in meningioma surgery.

3. Navigation

**Fig. 6:**
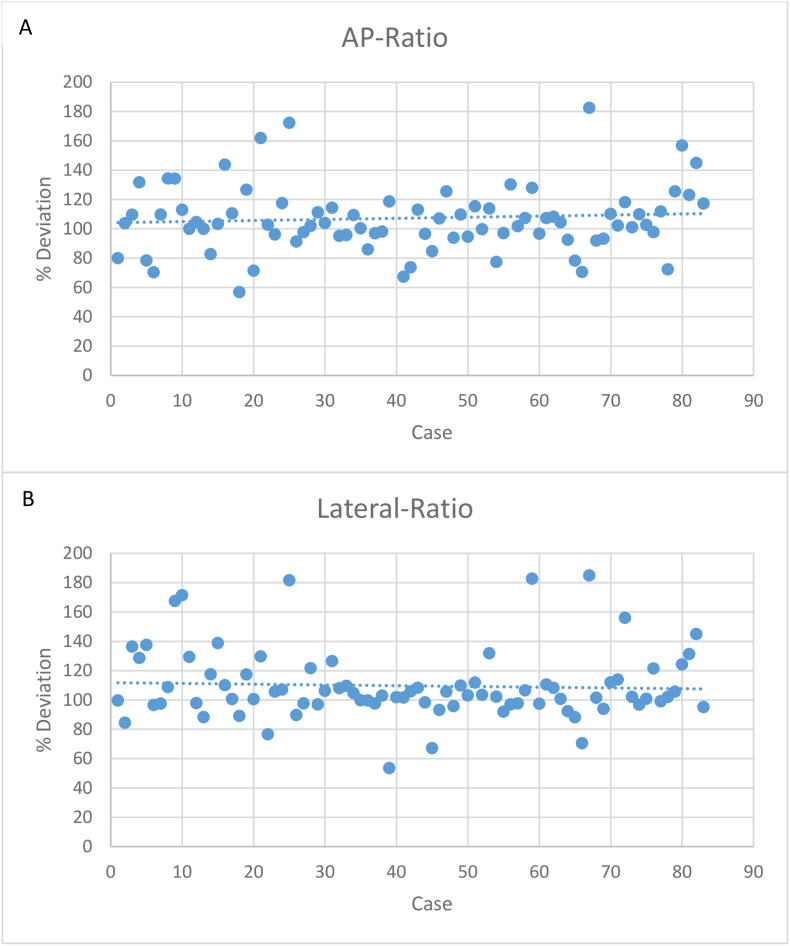
Transfer from preoperative iVR-planing to intraoperative neuronavigation using the brainlab navigation system depicting the meningioma (red), corticospinal tract (blue) and arcuate fasciculus (green). A: iVR-planning with adjusted positioning and marked skin incision, craniotomy and dural opening; intraoperative AR-overlay, marked skin incision (B) and post-craniotomy (C).

As proposed by Zhou et al. (2022), mixed reality might be a feasible tool for navigating emergency minimally invasive procedures, where extensive brain exposure might be replaced by minimal invasive techniques, even in highly eloquent areas (Tang et al., 2024). As a guidance tool, it might also enhance the safety and precision in anatomically challenging scenarios (Vychopen et al., 2024). Neuronavigation is currently performed primarily using the Brainlab navigation system. When iVR planning has been carried out using the Brainlab-suite, the idenitcal 3D volumes of anatomical and pathological structures, as well as depiction of fiber tracts or trajectories, can be displayed via the navigation pointer or the microscope's head-up display (see Fig. 6). This seamless transfer from simulation to the operating room ensures a continuous integration of preoperative planning and intraoperative guidance, representing a key element for the value of preoperative iVR planning. In the future, swift surface registration with 3D model of the pathology might change the way of treatment due to swift availability of precise trajectory planning, allowing the surgeon to minimize the collateral damage in emergency setting.

A relevant limitation of this study is the use of the AP/lateral ratio instead of absolute measurements. This approach was necessitated by the use of different iVR-platforms and may represent a source of projection-related measurement inaccuracy. Future studies should prioritize the use of a single presentation platform. Also, differences in measurements among assessors were resolved by consensus. For follow-up studies, reproducibility should be improved through the use of a predefined measurement protocol and a formal interobserver reliability assessment.

As a tool for simulation of meningioma surgery, iVR fills the gap between text-book based learning and hands-on training, as it combines anatomical and pathological reality in an interactive interface. This simulation allows safe exploration of various surgical options, while always respecting the individual patient's anatomy. We believe that an application of iVR planning into daily routine may significantly contribute to accelerate the learning curve of students and residents, while simultaneously increasing the patients' safety by improving awareness of physiological and distorted anatomy and thus creating a deeper understanding of the surgical procedure. Carefully planned and meticulously performed approaches are vital for optimal results for the resection of meningioma. We are confident that preoperative iVR-evaluation and planning will represent a key aspect of meningioma surgery in the near future.

## 5 Conclusion

Operative planning using 3D iVR-models is capable of depicting the operative anatomical reality with a high degree of accuracy. iVR is an increasingly important tool in planning and training in meningioma surgery and may become an integral part of everyday surgical practice in the near future.

## Compliance with ethical standards

The manuscript has been prepared in accordance with the instructions provided by the authors and has been approved by all of them. All of the requirements set out by the authors have been met and approved by all of them. The authors have no conflicts of interest to declare. All co-authors have seen and agree with the content of the manuscript and there is no financial interest to report. We certify that the submission is original work and is not under review at any other publication.

## Human ethics and consent to participate declarations

Not applicable, as this is a retrospective study and no additional patient data was raised.

## Institutional review board statement

The study was conducted in accordance with the Declaration of Helsinki. IRB approval was obtained by the local ethics committee of the University of Leipzig, medical faculty under the chairmanship of Prof. Dr. Dr. Ortrun Riha (No.: 344/25-ek).

## Declaration of generative AI and AI-assisted technologies in the manuscript preparation process

During the preparation of this work the author(s) did not use AI and AI-assisted technologies, besides potential AI-based technologies implemented in the brainlab suite.

## Funding

No funding was received for this research.

## Declaration of competing interest

The authors declare the following financial interests/personal relationships which may be considered as potential competing interests: Sebastian Lehmann reports was provided by Uniklinikum Leipzig. Se reports a relationship with University Hospital Leipzig that includes: employment. If there are other authors, they declare that they have no known competing financial interests or personal relationships that could have appeared to influence the work reported in this paper.
